# Time perception, phonological skills and executive function in children with dyslexia and/or ADHD symptoms

**DOI:** 10.1111/j.1469-7610.2010.02312.x

**Published:** 2011-02

**Authors:** Debbie Gooch, Margaret Snowling, Charles Hulme

**Affiliations:** University of YorkUK

**Keywords:** Dyslexia, attention deficit/hyperactivity disorder, comorbidity, attention, time perception, executive function, phonological skills

## Abstract

**Background:**

Deficits in time perception (the ability to judge the duration of time intervals) have been found in children with both attention-deficit/hyperactivity disorder (ADHD) and dyslexia. This paper investigates time perception, phonological skills and executive functions in children with dyslexia and/or ADHD symptoms (AS).

**Method:**

Children with dyslexia-only (*n* = 17), AS-only (*n* = 17), comorbid dyslexia+AS (*n* = 25), and typically developing controls (*n* = 42), matched for age and non-verbal ability, were assessed on measures of phonological skills, executive function and time perception (duration discrimination and time reproduction).

**Results:**

Children with dyslexia were impaired on measures of phonological skill and duration discrimination compared to children without dyslexia (though problems on duration discrimination appeared to be attributable to mild symptoms of inattention in this group). In contrast, children with AS exhibited impairments on measures of both time perception and executive function compared to children without AS. Children with dyslexia+AS showed an additive combination of the deficits associated with dyslexia-only and AS-only.

**Conclusions:**

Dyslexia and AS appear to be associated with distinct patterns of cognitive deficit, which are present in combination in children with dyslexia+AS.

Dyslexia and attention-deficit/hyperactivity disorder (ADHD) are both common childhood disorders. It is widely accepted that the proximal cognitive cause of dyslexia is a phonological deficit (Vellutino, Fletcher, Snowling, & Scanlon, 2004) whereas the predominant account of ADHD sees it as arising from an impairment in executive functions that affects both cognitive and motivational systems (Barkley, 1997). The frequent comorbidity of reading and attention difficulties raises the issue of whether common causal mechanisms, at either the cognitive or biological levels, may be involved (Light, Pennington, Gilger, & DeFries, 1995; Stevenson et al., 2005).

There are a number of competing explanations of the comorbidity between dyslexia and ADHD. In one of the first papers to address this issue, Pennington, Groisser, and Welch (1993) proposed that the symptoms of ADHD associated with dyslexia are a secondary consequence of reading problems (‘phenocopy’ hypothesis). However, later studies failed to support this hypothesis (e.g., Willcutt et al., 2001; Willcutt, Pennington, Olson, Chhabildas, & Hulslander, 2005), instead reporting that comorbid dyslexia+ADHD is associated with a combination of the cognitive impairments seen in dyslexia and ADHD alone (Adams & Snowling, 2001; Nigg, Hinshaw, Carte, & Treuting, 1998; Raberger & Wimmer, 2003; Rucklidge & Tannock, 2002; Willcutt et al., 2005).

A different view of the aetiology of comorbid dyslexia+ADHD is that the condition arises from shared genetic risk factors that contribute to the development of separate cognitive impairments that underlie the two disorders. In this view, those same genetic risk factors (acting in concert with other genetic and environmental risk factors) may lead to the development of both underlying cognitive impairments, in turn producing the comorbid condition (‘shared aetiology’ hypothesis; de Jong, Oosterlaan, & Sergeant, 2006). Arguably, the pattern of findings just described (that dyslexia-only and ADHD-only are associated with different patterns of cognitive impairment but both forms of impairment co-occur in the comorbid condition) is consistent with this hypothesis.

Finally, it has occasionally been argued that comorbid dyslexia+ADHD may reflect different causal mechanisms from those operating in either condition alone (‘cognitive subtype’ hypothesis, de Jong et al., 2006). Findings that children with dyslexia+ADHD show more severe impairments in phonological skills, inhibition (Willcutt et al., 2001), rapid automatised naming (Rucklidge & Tannock, 2002) and time estimation (McGee, Brodeur, Symons, Andrade, & Fahie, 2004) than children with either disorder alone have been interpreted as supporting this hypothesis.

There is currently growing interest in the search for the ‘endophenotypes’ underlying developmental disorders (Skuse, 2001). Endophenotypes can be defined as heritable processes, intermediate between the genotype and the behavioural phenotype that reflect an underlying liability for a disorder (Almasy & Blangero, 2001), which may combine with additional risk factors to lead to a clinical diagnosis (Bishop, 2006). On the basis of findings from a family risk study of dyslexia, Snowling (2008) proposed that phonological deficits may be better conceptualised as an endophenotype of dyslexia rather than a marker of reading difficulties per se, because some individuals with phonological deficits can compensate for these to become competent readers.

Castellanos and Tannock (2002) discuss a number of putative cognitive endophenotypes for ADHD, including deficits in inhibition, sustained attention, response variability, working memory and temporal processing. They suggest that temporal processing deficits may be causally related to deficits in time perception (see Toplak, Dockstader, & Tannock, 2006) and response variability (e.g., Kunsti & Stevenson, 2001) seen in ADHD. They also suggest that temporal processing deficits may underpin the deficits in time perception (Nicolson, Fawcett, & Dean, 1995) and phoneme awareness associated with dyslexia (although this remains controversial (Nittrouer, 1999; Marshall, Snowling, & Bailey, 2001)). According to this hypothesis, deficits in temporal processing may be an endophenotype of both ADHD and RD that could explain the frequent comorbidity between these disorders.

The present study aimed to clarify the comorbidity between dyslexia and ADHD by investigating the possible cognitive endophenotypes of these disorders in children with dyslexia and/or ADHD symptoms (AS). Children’s performance on measures of phonological skills, executive function and time perception were examined. According to the ‘phenocopy’ hypothesis, children with dyslexia+AS will only exhibit the cognitive deficits associated with dyslexia since their AS arise as a secondary consequence of reading problems. In contrast, the ‘cognitive subtype’ hypothesis suggests that the impairments observed in dyslexia+AS should be more severe than those observed in children with either dyslexia or AS alone. The ‘shared aetiology’ hypothesis suggests that children with dyslexia-only and AS-only will show distinct patterns of cognitive impairments and both forms of impairment will co-occur in the comorbid condition. Alternatively, if it could be demonstrated that deficits in time perception are associated with both dyslexia and AS, this would provide some evidence for a shared cognitive endophenotype underlying both conditions. Such a pattern would, in turn, give a possible explanation for how shared genetic risk factors might operate to cause these disorders and the comorbidity between them.

## Method

### Participants

Children with dyslexia and/or AS were recruited from a child and adolescent mental health services (CAMHS) department, the Centre for Reading and Language, Dyslexia Action and from schools in York, England. Typically developing (TD) children were recruited from the same schools as the children with dyslexia and with AS. Ethical clearance for this study was granted by the University of York, Department of Psychology, Ethics Committee and by the York, NHS Research Ethics Committee. Informed parental consent was obtained and the children completed consent forms prior to participating.

All children completed a screening assessment including the Matrices, Vocabulary, Word Reading and Spelling subtests from the *British Abilities Scales-II* (*BAS II;*Elliott et al., 1996) and their parents and teachers were asked to complete a rating scale for AS (e.g., Barkley & Murphy, 1998; Hulslander et al., 2004). Children were excluded if they obtained a below average non-verbal ability score (*t*-score < 40). None of the participating children had known neurological or sensory impairments. Children were assigned to groups according to the criteria outlined below. Those who met criteria for both dyslexia and had AS were assigned to the dyslexia+AS group. Participants who were receiving pharmacological treatment for ADHD (*n* = 9) were asked to discontinue their medication at least 24 hours prior to the research session.

#### Classification of dyslexia

Children who obtained standard scores of 85 or below on either the Word Reading or the Spelling scales were classified as having dyslexia (*n* = 42) and those with scores above 85 were considered to be normal readers (*n* = 59).

#### Classification of AS

The rating scale used to measure AS in this study contained statements pertaining to each of the 18 DSM-IV ADHD symptoms (see Appendix A for details, reliability and validation). Nine of the statements targeted symptoms of inattention (e.g., ‘Is good at sustaining attention on tasks or play activities’) and nine targeted symptoms of hyperactivity/impulsivity (e.g., ‘Runs about or climbs excessively in situations in which it is inappropriate’). Respondents were asked to rate the child’s behaviour over the past 6 months on a four-point scale from 0 (false) to 3 (true) for each statement. As in previous studies in which similar rating scales were used (e.g., Willcutt et al., 2005), ratings of 2 or 3 were deemed clinically significant and thus scored as a positive symptom (scoring was reversed for positively worded items). Completed questionnaires were returned from both parents and teachers for 52 of the children, only teacher ratings were received for 39 of the children and only parent ratings were received for 10 of the children (see Appendix A, Table 2).

**Table 2:** Characteristics of the four groups

	TD-controls (*N* = 42)		Dyslexia-only (*N* = 17)		AS-only (*N* = 17)		Dyslexia+AS (*N* = 25)		*F*	*p*	*η*_*p*_^*2*^
Age	10.27_a_	(2.63)	10.69_a_	(1.77)	9.54_a_	(2.60)	10.33_a_	(1.42)	.79	.50	.02
Vocabulary (VIQ) ^1,2^	48.55_a_	(8.41)	46.53_a_	(3.86)	47.82_ab_	(7.87)	41.24_b_	(8.02)	5.09	.00	.14
Matrices (NVIQ) ^1,2^	52.95_a_	(5.97)	51.41_a_	(7.52)	51.82_a_	(6.17)	51.72_a_	(5.53)	.37	.77	.01
Word reading ^1,3^	108.81_a_	(12.37)	75.47_c_	(6.44)	101.53_a_	(12.41)	81.92_b_	(8.22)	57.15	.00	.64
Spelling ^1,3^	110.12_a_	(11.81)	79.18_c_	(8.99)	100.76_b_	(10.24)	75.28_c_	(6.48)	80.49	.00	.71

	(*N* = 10)^y^		(*N* = 15)^y^		(*N* = 14)^y^		(*N* = 23)^y^				
Parent rated inattention (9)	2.00_a_	(1.70)	3.07_a_	(1.71)	7.36_b_	(1.39)	6.70_b_	(1.79)	34.07	.00	.64
Parent rated hyperactivity/impulsivity (9)	1.10_a_	(1.20)	1.33_a_	(1.54)	5.14_b_	(3.37)	4.57_b_	(3.45)	8.09	.00	.30

	(*N* = 38)^z^		(*N* = 16)^z^		(*N* = 17)^z^		(*N* = 20)^z^				
Teacher rated inattention (9)	.82_a_	(1.27)	2.38_b_	(1.67)	4.71_c_	(2.80)	6.00_c_	(2.51)	34.84	.00	.55
Teacher rated hyperactivity/impulsivity (9)	.42_a_	(.79)	.81_a_	(1.05)	4.18_b_	(2.65)	3.15_b_	(2.60)	22.76	.00	.44

*Note*: ^1^ = subtests from BAS-II, ^2^ = t-scores, ^3^ = standard scores, ^y^ = subsample with parent ratings of AS, ^z^ = subsample with teacher ratings of AS. Means with common subscripts are not significantly different after Games–Howell correction for multiple comparisons (*p* < .05).

Children were classed as having AS if they had six or more symptoms in either the inattention or hyperactivity/impulsivity domain, or if they had more than six symptoms in both domains, as rated by either their parent or teacher: 44 children met criteria for AS; the majority were rated as inattentive (*n* = 22) or as showing deficits in both domains (*n* = 20). Only two children were rated as hyperactive/impulsive and given that the cognitive weaknesses associated with these symptoms are different from those associated with symptoms of inattention (e.g., Chhabildas, Pennington, & Willcutt, 2001), they were excluded from the analyses reported here.

Of the children with AS (*n* = 42), 15 had previously received a clinical diagnosis of ADHD combined-type, in the absence of any other comorbid diagnosis, by an experienced multidisciplinary CAMHS team. The remaining 27 children were recruited on the basis of substantial parental/teacher concerns about their attention and behaviour but did not have a clinical diagnosis. Comparison of the children with AS with and without a clinical diagnosis of ADHD showed that both performed within the average range for their age on measures of non-verbal IQ (NVIQ) and they did not differ significantly in terms of age, verbal IQ (VIQ), BAS reading or spelling scores. Furthermore, although children with a diagnosis of ADHD tended to be rated by parents as showing more symptoms of inattention and hyperactivity/impulsivity than children without a diagnosis, overall teacher ratings did not significantly differ between the groups and both groups were rated as having significantly more symptoms of ADHD than TD-controls (see Appendix B for details of this comparison).

None of the children with AS had diagnoses of other disruptive behaviour disorders, e.g., conduct disorder or oppositional defiant disorder; however, parents’ ratings of the children’s behaviour on the Strengths and Difficulties Questionnaire (SDQ; Goodman, 2005, 1997) (see Table 1) indicated that the majority of children with AS were rated as abnormal on the SDQ total difficulties score. In comparison, only 13% of the children with dyslexia-only were rated as abnormal. Furthermore, more children with AS were rated as abnormal on the hyperactivity and conduct problems subscales of the SDQ compared to children with dyslexia-only. These findings are in line with research that suggests high comorbidity between ADHD and disruptive behaviour disorders (e.g., Rommelse et al., 2009).

**Table 1:** The number of children with dyslexia-only, AS-only and dyslexia+AS who were classified as ‘abnormal’ on the Strengths and Difficulties Questionnaire (SDQ) subscales

	Dyslexia-only (*N* = 15)		AS-only (*N* = 14)		Dyslexia+AS (*N* = 23)			
					
	*N*	%	*N*	%	*N*	%	χ^2^	*p*
Emotional control	3	20.0	7	50.0	11	47.8	3.66	.16
Conduct problems	1	6.7	8	57.1	10	43.5	8.81	.01
Hyperactivity	1	6.7	10	71.4	14	60.9	14.87	.00
Peer problems	2	13.3	5	35.7	8	34.8	2.48	.29
Prosocial behaviour	0	0.0	4	28.6	1	4.3	8.12	.02
SDQ total difficulties	2	13.3	9	64.3	13	56.5	9.35	.01

#### TD controls

These children performed within the normal range for their age on the BAS Matrices, Word Reading and Spelling scales (standard scores greater than 85) and did not meet criteria for AS as rated by teachers or parents. Teachers also confirmed the absence of any difficulties with attention, behaviour or learning.

#### Sample characteristics

Four groups of children aged between 5.58 years and 14.75 years (mean = 10.23, *SD* = 2.24) participated in the study: 17 with dyslexia-only (5 female), 17 with AS-only (6 female), 25 with dyslexia+AS (5 female) and 42 TD controls (23 female). Table 2 shows that the groups did not differ in age or non-verbal ability but children with dyslexia+AS obtained lower Vocabulary scores than controls. As expected, children with dyslexia (dyslexia-only and dyslexia+AS) had lower reading and spelling scores than children without dyslexia (AS-only and TD-controls). In addition, children with AS (AS-only and dyslexia+AS) were rated by both teachers and parents as having more symptoms of ADHD than children without AS (dyslexia-only and TD-controls). Importantly, children with dyslexia+AS did not exhibit more severe reading difficulties than children with dyslexia-only or more symptoms of ADHD than the AS-only group. Thus the co-occurrence of dyslexia and AS was not confounded with severity. A further finding of note was that, in relation to TD-controls, children with AS-only performed significantly worse in spelling and children with dyslexia-only exhibited significantly more symptoms of inattention, as rated by their teachers. These findings suggest that the children with dyslexia-only and AS-only exhibited mild symptoms of the other disorder, even though they did not meet our criteria for classification.

### Tests and procedures

Each child completed tasks tapping phonological skills, executive function and time perception in a fixed order over 2–4 sessions.

#### Phonological skills

Phonological memory was assessed using the *Children’s Nonword Repetition task* (CNRep; Gathercole & Baddeley, 1996), in which the child had to repeat 40 multi-syllabic nonsense words, and *Digit Recall* from the *Working Memory Test Battery for Children* (WMTB-C; Pickering & Gathercole, 2001), in which the child heard a list of numbers and had to repeat them back in the same order.

Phoneme awareness was assessed with a task in which the child had to delete a specified phoneme from a spoken nonword (McDougall, Hulme, Ellis, & Monk, 1994). There were 24 items, and deletion of the specified phoneme always resulted in a word, e.g., deleting the /b/ from the nonword /beis/ produces the word *ice*.

Phonological decoding (nonword reading) was measured using the *Phonemic Decoding* subtest from the Test of Word Reading Efficiency (TOWRE; Torgesen, Wagner, & Rashotte, 1999).

#### Executive function

Behavioural inhibition was assessed using a computerised *Stop Signal Reaction Time (SSRT)* task adapted from Logan, Schachar, and Tannock (1997). The primary *go*-task was a visual choice reaction time task; a 500ms central fixation point was followed by the presentation of either the letter *x* or the letter *o*. On 25% of the trials a stop-signal (100ms, 1000Hz auditory tone) was presented shortly after the *x* or *o* appeared on the screen, informing the participant that they must inhibit their response to the *go*-task on that trial (*stop*-task). The children were instructed to press the letter on the keyboard corresponding to the letter on the screen as quickly as possible but to try to stop their response if they heard the stop-signal.

The delay between the presentation of the visual stimulus and the onset of the stop-signal (stop-signal delay (SSD)) was initially set to 250ms and was adjusted up or down in 50ms increments depending on the accuracy of the child’s response (Logan et al., 1997). This procedure converged on the SSD at which the child failed to inhibit on 50% of the trials. The mean probability of responding (not inhibiting) on a stop-signal trial was .52, indicating that the tracking algorithm succeeded.

After completing two blocks of practice trials (24 *go*-task trials followed by the random presentation of 18 *go-* and 6 *stop-*trials) with feedback on accuracy and speed, the children completed three blocks of trials each comprising 30 *go*-task trials interspersed with 10 *stop*-task trials. Each child’s mean reaction time (MRT) and standard deviation of reaction time (SDRT; a measure of variability in responding) were calculated from correct *go*-task trials. SSRT (a measure of inhibition) was estimated for each child by subtracting their mean SSD from their MRT, providing an index of the duration of the inhibitory process, independent of mean reaction time. A long SSRT is indicative of poorer inhibition.

Given the findings of visuo-spatial memory deficits in ADHD (e.g., Savage, Cornish, Manley, & Hollis, 2006; de Jong et al., 2009; Rhodes, Coghill, & Matthews, 2005), *Block Recall* from the WMTB-C (Pickering & Gathercole, 2001) was used to assess visuo-spatial memory; here the child saw the examiner tap a sequence of blocks on a *Block Recall* board and then recalled the sequence by tapping the blocks in the same order. In addition, *Listening Recall* from the WMTB-C was used to assess working memory processes; here the child heard a list of sentences and was asked to decide whether each sentence was true or false before recalling the last word from each sentence.

Sustained attention was assessed using the *Score!* subtest from the *Test of Every Day Attention for Children* (TEACh; Manly, Robertson, Anderson, & Nimmo-Smith, 1999). In this task the child silently counted the number of ‘scoring’ sounds they heard. There were 10 trials, each with between 9 and 15 ‘scoring’ sounds. The number of trials where the child correctly counted the ‘scoring’ sounds was recorded.

#### Time perception

Each child completed computerised duration discrimination and time reproduction tasks.

*Duration discrimination.* This task was adapted from that used by Nicolson et al. (1995) and Ramus, Pidgeon, and Frith (2003)*.* As recommended by Halliday and Bishop (2006), a three-interval three-alternative forced choice (oddball) paradigm was used to reduce memory load. On each trial, the child heard three 1000Hz tones, two of which were 1200ms long and a roving target which was a different length (400ms, 700ms, 800ms, 900ms, 1000ms and 1100ms – each repeated nine times). The child was required to decide which tone was the ‘odd one out’. Six easy ‘catch-trials’ consisting of two 1500ms tones and one 200ms tone were interspersed within the experimental trials. The proportion of errors made on these trials was used to monitor attention to the task.

Six practice trials with feedback were followed by 60 experimental trials, with short breaks between each block of 10 trials. The percentage of correct responses was plotted against the six target durations for each child and linear regression was used to estimate a threshold (the point at which the child could discriminate the shorter target tone from the standard tone with 75% accuracy).

*Time reproduction.* Children were required to reproduce the duration of a visually presented target (2, 4, 6, 8 and 10s). The child was instructed to watch a blue square and then prompted to hold down the spacebar to make a red square appear for the same length of time. Each target time interval was presented twice and the duration reproduced by the child was recorded. An absolute error score for each target duration was calculated by taking the average value of the magnitude of the discrepancy between the child’s time reproduction and the target duration for each target interval.

## Results

### Data treatment

Three children with AS-only, 5 with dyslexia+AS and 3 TD-controls displayed extreme performance on the duration discrimination (either because of very good or very poor performance). To improve the shape of the distribution while preserving the rank order of the outlying scores (±3 SD or more from the group mean), each was replaced by a value equal to the next highest non-outlying score plus one unit of measurement (Winzorisation; Tabachnick & Fidell, 2001).

Means and standard deviations describing children’s performance on measures of phonological skills, executive function and time perception are presented in Table 3. To determine whether dyslexia or AS was significantly associated with poorer performance on any of the cognitive measures, independent of the other disorder, a series of 2 × 2 (dyslexia(+/-) × AS(+/-))ANOVAs were conducted. A significant main effect of dyslexia indicates that children with dyslexia are performing worse on the measure compared to children without dyslexia; a significant main effect of AS indicates that children with AS are performing worse on the measure than children without AS; the absence of an interaction suggests that the effects of dyslexia and AS are statistically independent of each other (additive). Planned comparisons, using independent sample *t*-tests, were conducted to test the ‘cognitive-subtype’ hypothesis that children with dyslexia+AS would show greater deficits than children with dyslexia-only or AS-only.

**Table 3:** Means and standard deviations for the measures of phonological skill, executive function and time perception for each of the four groups; main effects and interactions from the 2×2 ANOVA are also displayed

	TD-control			Dyslexia-only			AS-only			Dyslexia+AS			Main effect				Interaction	
						
													Dyslexia		AS		Dyslexia × AS	
															
Measure	*M*	*SD*	*n*	*M*	*SD*	*n*	*M*	*SD*	*n*	*M*	*SD*	*n*	*F*	*η*_*p*_^*2*^	*F*	*η*_*p*_^*2*^	*F*	*η*_*p*_^*2*^
Phonological skills																		
Phoneme deletion (18)	13.31	3.80	42	10.53	4.09	17	11.94	4.13	17	9.76	3.59	25	9.11^**^	.09	1.69	.02	.13	.00
CNRep (40)	35.37	3.69	35	30.94	5.15	17	32.71	5.76	17	31.36	3.77	25	9.15^**^	.09	1.38	.02	2.61	.03
Phonemic decoding (63)	37.82	14.44	38	15.12	9.05	17	29.71	19.73	17	18.76	9.77	25	32.62^**^	.26	.58	.01	3.89^*^	.04
Digit recall (54)	29.98	4.42	40	26.47	4.23	17	39.00	6.59	17	27.84	4.14	25	5.26^*^	.05	.04	.00	1.33	.01
Executive function																		
Inhibition (SSRT task)																		
MRT (ms)	653.97	172.58	35	663.72	142.19	17	726.93	174.30	17	685.42	171.46	24	.19	.00	1.70	.02	.50	.01
SDRT (ms)	204.65	83.63	35	200.84	60.48	17	257.96	85.86	17	249.10	68.24	24	.15	.00	9.37^**^	.10	.02	.00
SSRT (ms)	316.64	103.96	35	336.47	80.10	17	372.22	146.61	17	369.93	126.05	24	.12	.00	3.18	.04	.20	.00
SSD (ms)	337.33	135.05	35	361.06	160.57	17	354.71	173.90	17	315.49	190.15	24	.05	.00	.16	.00	.80	.01
Probability of inhibition	.54	.08	35	.54	.10	17	.53	.14	17	.51	.12	24	.12	.00	.86	.01	.02	.00
Memory skills																		
Listening recall (36)	12.18	3.02	39	12.94	3.78	17	10.59	5.09	17	11.08	3.57	25	.62	.01	4.72^*^	.05	.03	.00
Block recall (54)	26.59	4.82	37	25.65	3.77	17	23.41	3.64	17	25.04	4.72	25	.13	.00	3.95^*^	.04	1.83	.03
Sustained attention																		
Score! raw score (10)	7.43	2.34	35	7.06	2.14	17	5.69	2.87	16	5.72	2.41	25	.10	.00	8.52^**^	.09	.15	.00
Time perception																		
Duration discrimination threshold (ms)	399.06	203.79	39	477.36	109.16	17	466.21	232.78	17	584.03	226.93	25	5.10^*^	.05	4.00^*^	.04	.21	.00
*‘*Catch-trial’ errors	.02	.06	39	.01	.04	17	.10	.17	17	.12	.14	25	.05	.00	15.94^**^	.15	.51	.01
TTRE (ms)	1336.45	1036.55	35	1287.89	755.67	16	1621.76	926.83	17	2064.17	998.05	25	.88	.01	6.38^*^	.07	1.37	.02

*Note*: TTRE = total time reproduction error; SSRT = stop signal reaction time; SSD = stop signal delay; MRT = mean reaction time; SDRT = standard deviation of reaction time, ^*^p < .05, ^**^p < .01.

### Phonological skills

As expected, there were significant main effects of dyslexia but not AS on all the measures of phonological skill (phoneme deletion, nonword repetition, phonemic decoding and digit recall), and planned comparisons revealed that the dyslexia+AS group performed similarly to the dyslexia-only group on all measures of phonological skill (all *t* < 1.24, *p* > .22). On only one task (phonemic decoding) was there a significant dyslexia × AS interaction which appears to reflect the fact that the comorbid dyslexia+AS group actually perform slightly, but non-significantly, better than the dyslexia-only group on this task.

### Executive function

As expected, significant main effects of AS were found on measures of response variability (SDRT), verbal working memory (listening recall), visuo-spatial memory (block recall) and sustained attention (Score!). There were also non-significant trends for children with AS to show longer SSRTs (reflecting weaker inhibition) compared to children without AS. Planned comparisons revealed that the dyslexia+AS group performed similarly to the AS-only group on all of the measures of response execution and executive function (all *t* < 1.26, *p* > .22) and worse than the dyslexia-only group on the measure of response variability (SDRT: *t* (39) = 2.39, *p* < .05). There was also a strong trend for the dyslexia+AS group to perform worse than the dyslexia-only group on the Score! test (*t* (40) = 1.89, *p* = .07; *d* = .59).

### Time perception

Children with dyslexia-only and AS-only performed similarly and less well than TD-controls on the duration discrimination task. There were significant main effects of dyslexia and AS but no significant interaction. Children with dyslexia+AS performed similarly to children with AS-only on this task (*t* (40) = 1.63, *p* = .11) but obtained significantly worse duration discrimination thresholds than children with dyslexia-only (*t* (40) = 2.03, *p* < .05). Children with AS made significantly more errors on the duration discrimination ‘catch-trials’ compared to children without AS. Children with dyslexia+AS made significantly more errors than children with dyslexia-only (*t* (16) = 3.70, *p* < .01) but performed similarly to children with AS-only on this measure of attention to task (*t* (40) = .43, *p* = .67).

To assess whether either dyslexia or AS was significantly associated with poorer performance on the time reproduction task as a function of target duration, children’s absolute error scores for each duration were entered into a mixed ANOVA (dyslexia × AS × target duration). The magnitude of absolute errors increased as the target duration increased (*F* (2.86, 254.45) = 39.78, *p* < .01, *η*_*p*_^*2*^ = .31) but none of the interactions between target duration and either dyslexia or AS were significant (all *F*s < 1). Data were therefore collapsed across durations to form a Total Time Reproduction Error (TTRE) score, shown in Table 3.

A significant main effect of AS confirmed that children with AS made larger absolute errors than children without AS. There was no significant main effect of dyslexia and no significant interaction. Children with dyslexia+AS made significantly larger absolute errors compared to children with dyslexia-only (*t* (15) = 2.82, *p* < .01) but performed similarly to children with AS-only on the time reproduction task (*t* (40) = 1.47, *p* = .15).

### Dimensional analyses

Duration discrimination deficits have been found to be associated with both dyslexia and AS in this and in previous studies (Nicolson et al., 1995; Toplak et al., 2006). To examine whether this weakness in the ability to discriminate small differences in duration could potentially be explained by symptoms of one disorder over and above symptoms of the other disorder, children’s duration discrimination thresholds were predicted from measures of dyslexia and AS.

Given the high correlation between children’s *BAS-II Word Reading* and *Spelling* scores (*r* = .91), a composite ‘literacy difficulty’ score was derived by averaging the standard scores for these measures. The number of inattentive and hyperactive/impulsive symptoms associated with the child’s highest overall ADHD rating was used as a measure of AS (ratings were not always available from both sources thus symptom ratings could not be combined).

Table 4 displays the results of a regression analysis predicting performance on the duration discrimination task (*n* = 98 children who completed this task). Together measures of inattention, hyperactivity and composite literacy difficulty scores accounted for 11% of the variance in duration discrimination thresholds. However, inattention was the only unique predictor (after controlling for hyperactivity and literacy; unique *r*^2^ = .05; *p* < .05) and neither hyperactivity/impulsivity nor literacy predicted additional unique variance (*r*^2^ = .00 and .01, respectively). This analysis suggests that performance on the duration discrimination task is related to problems of inattention rather than literacy difficulties.

**Table 4:** Simultaneous regression analysis predicting duration discrimination thresholds from continuous measures of dyslexia and ADHD symptomatology

	β	*t*	Unique *R*^*2*^
Symptoms of inattention	.33	2.20^*^	.05
Symptoms of hyperactivity	−.10	−.71	.00
Literacy Difficulties^1^	−.12	−1.07	.01
Overall *R*^*2*^	.11		

1 Literacy difficulties is a composite score created by averaging BAS reading and spelling standard scores; ^*^ = *p* < .05.

## Discussion

We compared children with dyslexia-only, AS-only, and dyslexia+AS on tests of phonological skills, executive function and time perception. Children with dyslexia-only and AS-only showed distinct cognitive profiles. Dyslexia was associated with deficits in phonological skills (phonological awareness, phonological memory and decoding) whereas AS was associated with increased response variability, deficits in attention and in visuo-spatial short-term memory (STM) (see also, Raberger & Wimmer, 2003; Rhodes et al., 2005; Willcutt et al., 2001). Children with AS made large errors on the time reproduction task and showed deficits in duration discrimination. Slightly poor duration discrimination also characterised children with dyslexia, probably reflecting mild impairments of attention in this group. Finally, on all measures, children with dyslexia+AS exhibited an additive combination of the deficits associated with the ‘pure’ conditions (dyslexia-only and AS-only).

Consistent with our hypotheses, children with dyslexia showed weaknesses on measures of phoneme deletion, nonword repetition, nonword reading and phonological memory. These measures are sensitive to deficits at the level of segmental phonemic representations, which have been viewed as a proximal cognitive cause dyslexia (Vellutino et al., 2004). In contrast, our findings regarding AS are consistent with findings of reaction time variability and deficits in visuo-spatial memory in ADHD (e.g., Castellanos & Tannock, 2002; Kunsti & Stevenson, 2001). The response variability associated with AS may reflect a difficulty in sustaining attention or cognitive activation (Sergeant, 2000).

This study included just one measure of visuo-spatial memory (Block Recall) and our findings of a deficit on this task are consistent with previous findings that ADHD is associated with deficits in visuo-spatial storage (Rhodes et al., 2005); it is unfortunate that our study did not include other possibly more attentionally demanding visuo-spatial memory tasks which have shown even larger deficits in children with ADHD (Martinussen, Hayden, Hogg-Johnson, & Tannock, 2005).

We evaluated three different views concerning the comorbidity of dyslexia and ADHD. Since children with dyslexia+AS performed poorly on measures of executive function as well as on phonological tasks, our findings refute the ‘phenocopy’ hypothesis (Pennington et al., 1993). They also failed to provide support for the ‘cognitive subtype’ hypothesis (e.g., de Jong et al., 2006; Rucklidge & Tannock, 2002) because children with dyslexia+AS exhibited neither different, nor more severe, deficits than children with either pure condition. Rather, our results suggest that the comorbid form reflects the effects of independent underlying cognitive causes (independent endophenotypes; Willcutt et al., 2001, 2005).

Although deficits in time reproduction were associated with AS and not dyslexia, deficits in duration discrimination were associated with both AS and dyslexia. However, the problems in duration discrimination appeared to reflect symptoms of inattention among the children with dyslexia rather than literacy difficulties per se. This finding is inconsistent with the hypothesis of Castellanos and Tannock (2002) that problems in temporal processing underpin the deficits in phonological awareness associated with dyslexia.

It is important to note that the lack of a diagnostic assessment of ADHD, which includes evidence of persistence and impairment, is a limitation in this study and may restrict the generalisability of the results to other ADHD samples. However, our results are generally consistent with those of previous research and highlight the need to consider the impact of comorbid symptoms of ADHD when investigating neuropsychological functioning in children with dyslexia. Overall our findings are consistent with the claim that dyslexia and symptoms of ADHD are the products of different cognitive deficits that may arise from shared genes with pleiotropic effects (Willcutt et al., 2001).

**Key points**

- Dyslexia and AS are associated with distinct cognitive profiles.
- Children with comorbid dyslexia+AS exhibit an additive combination of the cognitive deficits associated with dyslexia-only and AS-only.
- Deficits in duration discrimination associated with dyslexia are mediated by symptoms of inattention resulting from the comorbidity between these disorders.
- It is important to consider the impact of ADHD symptoms when investigating the neuropsychological profile of children with dyslexia.

## Supplementary material

The following supplementary material is available for this article:

**Appendix A.** Details, reliability and validation of the rating scale used to measure the classification of AS (Word document)

**Appendix B.** Children with ADHD symptoms (AS) with and without a clinical diagnosis of ADHD (Word document)

This material is available as part of the online article from:

http://www.blackwell-synergy.com/doi/abs/10.1111/j.1469-7610.2010.02312.x
