# Supplementary material for: Time perception, phonological skills and executive function in children with dyslexia and/or ADHD symptoms
Source: J Child Psychol Psychiatry. 2011 Feb;52(2):195–203. doi: 10.1111/j.1469-7610.2010.02312.x (PMC3412207; doi:10.1111/j.1469-7610.2010.02312.x)
Supplement: Supplementary file 1 [file jcpp0052-0195-SD1.doc]

[app]**Appendix A** Details, reliability and validation of the rating scale used to measure the classification of AS

[tc]**Table 1** Items from the ADHD rating scale used to classify children as having ADHD symptoms (AS)

| Hyperactivity/impulsivity | |
| --- | --- |
| H1 | Interrupts or intrudes on others (e.g., butts into conversations or games) |
| H2 | Is often ‘on the go’ or acts as if driven by a motor |
| H3 | Can play or take part in leisure activities quietly |
| H4 | Runs about or climbs excessively in situations in which it is inappropriate |
| H5 | Talks all the time or often too much |
| H6 | Fidgets with hands or taps feet |
| H7 | Is good at waiting their turn in games/activities |
| H8 | Leaves seat in classroom or in other situations in which remaining seated is expected |
| H9 | Blurts out answers before questions have been completed |
| Inattention | |
| I1 | Does not seem to listen to when spoken to directly |
| I2 | Forgetful in daily activities |
| I3 | Always follows through on instructions and finishes school work or chores |
| I4 | Loses things necessary for tasks or activities (e.g., toys, school assignments, pencils, books or tools) |
| I5 | Is good at sustaining attention in tasks or play activities |
| I6 | Is easily distracted from tasks by background noise or activity |
| I7 | Fails to give close attention to detail |
| I8 | Is good at organising tasks and activities |
| I9 | Dislikes or is reluctant to engage in tasks that require sustained mental effort (e.g., school work or homework) |

[tc]**Table 2** The number of ADHD rating scales returned by each respondent for each of the four groups

|  | TD-Controls  (*N* = 42) | Dyslexia-only  (*N* = 17) | AS-only  (*N* = 17) | Dyslexia+AS  (*N* = 25) |
| --- | --- | --- | --- | --- |
| Parents | 4 | 1 | 3 | 5 |
| Teachers | 32 | 2 | – | 2 |
| Both | 6 | 14 | 14 | 18 |

[b]*Reliability of the ADHD rating scale*

[txt]Cronbach’s alphas for parent and teacher ratings of ADHD symptoms in the current sample were .95 and .94 respectively. This level of internal consistency is similar to that found by other research groups (e.g., DuPaul, Barkley, & Connor, 1998; Hulslander et al., 2004). Inter-rater reliability based on the subsample of children for whom parent and teacher ratings were available (*n* = 52) was found to be good in comparison to previous studies (McGrath et al., 2007); overall ADHD symptom ratings correlated at *r* =.53, inattentive symptom ratings correlated at *r* =. 39 and hyperactive-impulsive symptom ratings correlated at *r* = .66.

[b]*Validity of the ADHD rating scale*

[txt]Table 3 displays the correlations between children’s ratings on the ADHD rating scale (ratings consist of the number of inattentive and hyperactive/impulsive symptoms associated with the child’s highest overall ADHD rating as well as the total number of ADHD symptoms) and the Strengths and Difficulties Questionnaire (SDQ; Goodman, 2005). There is a strong correlation between the total number of ADHD symptoms and the SDQ hyperactivity rating, suggesting that these two scales measure similar constructs. The correlation between the SDQ hyperactivity rating and ratings of symptoms of hyperactivity/impulsivity is also strong. The relationships between SDQ subscales and the ratings of ADHD inattention are somewhat weaker, reflecting the fact that few of the SDQ items target behaviours associated with inattention.

[tc]**Table 3** Correlations between children’s ADHD symptom ratings and scales from the Strengths and Difficulties Questionnaire (SDQ) (*n* = 56)

|  | 1 | 2 | 3 | 4 | 5 | 6 | 7 | 8 |
| --- | --- | --- | --- | --- | --- | --- | --- | --- |
| 1. Inattention |  |  |  |  |  |  |  |  |
| 2. Hyperactivity/Impulsivity | .731_a_^**^ |  |  |  |  |  |  |  |
| 3. Total ADHD | .933_a_^**^ | .923_a_^**^ |  |  |  |  |  |  |
| 4. Total difficulties score (SDQ) | .608^**^ | .734^**^ | .762^**^ |  |  |  |  |  |
| 5. Emotional difficulties (SDQ) | .404^**^ | .379^**^ | .432^**^ | .768^**^ |  |  |  |  |
| 6. Conduct problems (SDQ) | .553^**^ | .712^**^ | .718^**^ | .885^**^ | .550^**^ |  |  |  |
| 7. Hyperactivity (SDQ) | .588^**^ | .771^**^ | .780^**^ | .856^**^ | .476^**^ | .765^**^ |  |  |
| 8. Peer problems (SDQ) | .468^**^ | .562^**^ | .587^**^ | .816^**^ | .545^**^ | .623^**^ | .569^**^ |  |
| 9. Prosocial (SDQ) | –.492^**^ | –.367^**^ | –.486^**^ | –.458^**^ | –0.183 | –.546^**^ | –.399^**^ | –.394^**^ |

[tn]_a_ = Subsample with ADHD ratings (*n* = 101).

[ref]**References**

DuPaul, G.J., Barkley, R.A., & Connor, D.F. (1998). Stimulants. In R.A. Barkley (Ed.), *Attention deficit hyperactivity disorder: A handbook for diagnosis and treatment* (pp. 510–551). New York: Guilford.

Goodman, R. (2005). *Strengths and Difficulties Questionnaire*. Retrieved November 1, 2005 from http://www.sdqinfo.com/questionnaires/english/c1.pdf.

Hulslander, J., Talcott, J., Witton, C., DeFries, J., Pennington, B., Wadsworth, S., Willcutt, E., & Olson, R. (2004). Sensory processing, reading, IQ, and attention. *Journal of Experimental Child Psychology*, *88*, 274–295.

McGrath, L.M., Hutaff-Lee, C., Scott, A., Boada, R., Shriberg, L.D., & Pennington, B.F. (2007). Children with comorbid speech sound disorder and specific language impairment are at increased risk for attention-deficit/hyperactivity disorder. *Journal of Abnormal Child Psychology*, *36*, 151–163.
