# Supplementary material for: Time perception, phonological skills and executive function in children with dyslexia and/or ADHD symptoms
Source: J Child Psychol Psychiatry. 2011 Feb;52(2):195–203. doi: 10.1111/j.1469-7610.2010.02312.x (PMC3412207; doi:10.1111/j.1469-7610.2010.02312.x)
Supplement: Supplementary file 2 [file jcpp0052-0195-SD2.doc]

[app]**Appendix B** Children with ADHD symptoms (AS) with and without a clinical diagnosis of ADHD

[txt]Twelve of the children who fulfilled criteria for AS had a diagnosis of ADHD combined-type from the CAMHS department in York. Three children were diagnosed with ADHD combined-type by clinicians outside the York area and 27 children did not have a clinical diagnosis of ADHD. Here we present a comparison of symptom severity, verbal and non-verbal ability and reading skills for children with AS with and without a formal diagnosis of ADHD.

*Symptom severity.* Data pertaining to the severity of ADHD symptoms in the children with AS who had a clinical diagnosis compared to the children without a clinical diagnosis (but who had significant attention difficulties) and TD-controls are presented in Tables 1 and 3. The data in Table 1 shows that children in the AS-only group who had a clinical diagnosis of ADHD are generally rated as having significantly more severe symptoms of ADHD by parents compared to children who do not have a clinical diagnosis. However, teachers rated children with AS-only with and without a clinical diagnosis of ADHD similarly in terms of the severity of their ADHD symptomatology. Post-hoc comparisons reveal that both groups were rated as showing significantly more symptoms of ADHD than TD-controls.

The data in Table 3 show that there is a trend for children in the dyslexia+AS group who had a clinical diagnosis of ADHD to be rated as having more severe symptoms of ADHD, by both parents and teachers, than the children who did not have a clinical diagnosis. However, post-hoc comparisons reveal that both groups were rated as showing significantly more symptoms of inattention and having significantly higher overall ADHD ratings than TD-controls.

*Age, IQ and reading skills***.** Tables 2 and 4 display the average age, VIQ, NVIQ, BAS reading and spelling scores for children with and without a clinical diagnosis of ADHD within the AS-only and dyslexia+AS groups. The data demonstrate that for both groups (AS-only and dyslexia+AS) there are few differences between the children with and without a clinical diagnosis of ADHD. The children with a diagnosis of ADHD in the dyslexia+AS group did, however, obtain significantly lower NVIQ scores compared to the children with dyslexia+AS who did not have a formal diagnosis, although both subgroups scored within the average range for their age.

[tc]**Table 1** Mean ADHD ratings from parents and teachers for children in the AS-only group with and without an ADHD diagnosis and TD-controls. Standard deviations are given in parentheses and the results from one-way ANOVA’s are also presented

|  | ADHD diagnosis | No diagnosis | TD-controls | *F* | *p* | *η_p_^2^* |
| --- | --- | --- | --- | --- | --- | --- |
|  | (*N* = 7)^y^ | ( *N* = 7)^y^ | ( *N* = 10) ^y^ |  |  |  |
| Parent ratings of inattention (max 9) | 8.29_a_ (.76) | 6.43_b_ (1.27) | 2.00_c_ (1.70) | 48.15 | .00 | .82 |
| Parent ratings of hyperactivity/impulsivity (max 9) | 8.00_a_ (1.83) | 2.29_b_ (1.50) | 1.10_b_ (1.20) | 47.51 | .00 | .82 |
| Parents ratings of ADHD (max 18) | 16.29_a_ (1.70) | 8.71_b_ (2.43) | 3.10_c_ (2.47) | 69.78 | .00 | .87 |
|  | ( *N* = 7)^z^ | ( *N* = 10) ^z^ | ( *N* = 38) ^z^ |  |  |  |
| Teacher ratings of inattention (max 9) | 4.29_ab_ (3.04) | 5.00_b_ (2.75) | .82_a_ (1.27) | 25.53 | .00 | .50 |
| Teacher ratings of hyperactivity/impulsivity (max 9) | 5.29_a_ (2.14) | 3.40_a_ (2.80) | .42_b_ (.79) | 38.71 | .00 | .60 |
| Teacher ratings of ADHD (max 18) | 9.57_a_ (4.79) | 8.40_a_ (5.25) | 1.24_b_ (1.72) | 36.36 | .00 | .58 |

[tn]^y^ = Subsample with parent ratings of ADHD, ^z^ = Subsample with teacher ratings of ADHD. Means with common subscripts are not significantly different after Games-Howell correction for multiple comparisons (*p* < .05).

[tc]**Table 2** Mean age, VIQ, NVIQ, BAS reading and spelling for children in the AS-only group with and without an ADHD diagnosis. Standard deviations are given in parentheses and the results from one-way ANOVA’s are also presented.

[tn]*Note*: ^1^ = subtests from the BAS-II, ^2^ = *t*-score, ^3^ = standard score, ^y^ = subsample with parent ratings of ADHD, ^z^ = subsample with teacher ratings.

|  | ADHD diagnosis  ( *N* = 7) | No diagnosis  ( *N* = 10) | *F* | *p* | *η_p_^2^* |
| --- | --- | --- | --- | --- | --- |
| Age | 10.24 (2.83) | 9.06 (2.46) | .84 | .37 | .05 |
| Vocabulary (VIQ) ^1, 2^ | 51.43 (9.98) | 45.30 (5.14) | 2.78 | .12 | .16 |
| Matrices (NVIQ) ^1, 2^ | 50.00 (8.66) | 53.10 (3.63) | 1.04 | .32 | .07 |
| Word reading ^1, 3^ | 106.43 (11.50) | 98.10 (12.40) | 1.97 | .18 | .17 |
| Spelling ^1, 3^ | 104.43 (8.44) | 98.20 (11.01) | 1.58 | .23 | .10 |

[tc]**Table 3** Mean ADHD ratings from parents and teachers for children in the dyslexia+AS group with and without an ADHD diagnosis and TD-controls. Standard deviations are given in parentheses and the results from one-way ANOVA’s are also presented

|  | ADHD diagnosis | No diagnosis | TD-controls | *F* | *p* | *η_p_^2^* |
| --- | --- | --- | --- | --- | --- | --- |
|  | ( *N* = 8)^y^ | ( *N* = 15)^y^ | ( *N* = 10) ^y^ |  |  |  |
| Parent ratings of inattention (max 9) | 7.50_a_ (1.07) | 6.27_a_ (1.98) | 2.00_b_ (1.70) | 27.26 | .00 | .65 |
| Parent ratings of hyperactivity/impulsivity (max 9) | 8.13_a_ (.99) | 2.67_b_ (2.66) | 1.10_b_ (1.20) | 30.12 | .00 | .67 |
| Parent ratings of ADHD (max 18) | 15.63_a_ (1.51) | 8.93_b_ (3.89) | 3.10_c_ (2.47) | 36.89 | .00 | .71 |
|  | ( *N* = 7) ^z^ | ( *N* = 13) ^z^ | ( *N* = 38) ^z^ |  |  |  |
| Teacher ratings of inattention (max 9) | 5.57_a_ (2.99) | 6.23_a_ (2.31) | .82_b_ (1.27) | 54.80 | .00 | .67 |
| Teacher ratings of hyperactivity/impulsivity (max 9) | 4.86_a_ (3.02) | 2.23_a_ (1.88) | .42_b_ (.79) | 29.45 | .00 | .52 |
| Teacher ratings of ADHD (max 18) | 10.43_a_ (5.59) | 8.46_a_ (3.73) | 1.24_b_ (1.72) | 49.69 | .00 | .64 |

[tn]^y^ = Subsample with parent ratings of ADHD, ^z^ = Subsample with teacher ratings of ADHD. Means with common subscripts are not significantly different after Games–Howell correction for multiple comparisons (*p* < .05).

[tc]**Table 4** Mean age, VIQ, NVIQ, BAS reading and spelling score for children in the dyslexia+AS group with and without an ADHD diagnosis. Standard deviations are given in parentheses and the results from one-way ANOVA’s are also presented

[tn]*Note*: ^1^ = subtests from BAS-II, ^2^ = *t*-score, ^3^ = standard score, ^y^ = subsample with parent ratings of ADHD, ^z^ = subsample with teacher ratings.

|  | ADHD diagnosis  ( *N* = 8) | No diagnosis  ( *N* = 17) | *F* | *p* | *η_p_^2^* |
| --- | --- | --- | --- | --- | --- |
| Age | 10.03 (2.11) | 10.48 (1.00) | .52 | .48 | .02 |
| Vocabulary (VIQ) ^1, 2^ | 38.38 (7.05) | 42.59 (8.28) | 1.54 | .23 | .06 |
| Matrices (NVIQ) ^1, 2^ | 48.25 (3.01) | 53.35 (5.74) | 5.51 | .03 | .19 |
| Word reading ^1, 3^ | 81.00 (8.54) | 82.35 (8.29) | .14 | .71 | .01 |
| Spelling ^1, 3^ | 73.88 (7.81) | 75.94 (5.91) | .54 | .47 | .02 |
